# A cluster of two human cases of tick-borne encephalitis (TBE) transmitted by unpasteurised goat milk and cheese in Germany, May 2016

**DOI:** 10.2807/1560-7917.ES.2018.23.15.17-00336

**Published:** 2018-04-12

**Authors:** SO Brockmann, R Oehme, T Buckenmaier, M Beer, A Jeffery-Smith, M Spannenkrebs, S Haag-Milz, C Wagner-Wiening, C Schlegel, J Fritz, S Zange, M Bestehorn, A Lindau, D Hoffmann, S Tiberi, U Mackenstedt, G Dobler

**Affiliations:** 1 Public Health Office, District of Reutlingen, Reutlingen, Germany; 2 State Health Office Baden-Württemberg, Stuttgart, Germany; 3 Veterinary Health Office, District of Reutlingen, Reutlingen, Germany; 4 Friedrich-Loeffler Institute, Federal Institute of Animal Health, Institute of Diagnostic Virology, Greifswald, Insel Riems, Germany; 5 Barts Health NHS Trust, London, United Kingdom; 6 Public Health Office, District of Biberach, Biberach, Germany; 7 Public Health Office, District of Sigmaringen, Sigmaringen, Germany; 8 Bundeswehr Institute of Microbiology; German Consultant Laboratory of TBE, German Center of Infection Research (DZIF), Munich, Germany; 9 Parasitology Unit, University of Hohenheim, Stuttgart, Germany

## Abstract

In May 2016, two cases of tick-borne encephalitis (TBE) were confirmed by serology (positive IgM and IgG antibodies against TBE virus (TBEV) in serum), with a possible link to raw milk and cheese from a goat farm in a region in Baden-Württemberg, Germany not previously known as TBE-endemic. The outbreak investigation identified 32 consumers of goat dairy products (29 consumers, one farm employee, two owners) of whom none had IgM antibodies against TBEV 3–8 weeks after consumption. Of the 27 notified TBE cases in the State, none reported consumption of raw goat milk or cheese from the suspected farm. Five of 22 cheese samples from 18 different batches were RT-qPCR-positive for TBEV -genome, and two of the five samples were confirmed by virus isolation, indicating viability of TBEV in the cheese. Nine of the 45 goats had neutralising TBEV antibodies, two of them with a high titre indicating recent infection. One of 412 *Ixodes ricinus* was RT-qPCR-positive, and sequencing of the E gene from nucleic acid extracted from the tick confirmed TBEV. Phylogenetic analyses of tick and cheese isolates showed 100% amino acid homology in the E gene and a close relation to TBEV strains from Switzerland and Austria.

## Introduction

Tick-borne encephalitis (TBE) is caused by an RNA virus of the genus *Flavivirus*, family *Flaviviridae* [1]. There are at least three known subtypes and so far, only the European subtype has been detected in various central European countries (Austria, Czech Republic, France, Germany, Slovak Republic, Switzerland) in ticks, rodents and patients since the 1950s [2]. TBE virus (TBEV) circulates between ticks and natural hosts (small mammals) in geographically strictly limited natural foci. TBE in animals is not well understood yet and the knowledge on the pathogenesis of TBEV in domestic animals is limited [3]. However, symptomatic infection has been reported in dogs, monkeys and horses, and seroconversion without specific neurological signs of TBE has been described in ruminants such as cattle, goats and sheep [4,5].

TBE in humans is the most important tick-borne viral disease in Europe and Asia, and up to 10,000 human cases are reported annually, although it is assumed that many mild and subclinical infections remain undiagnosed and both the infection and the disease are highly under-reported [6]. The main infection route of TBEV is by tick bite. However, food-borne transmission via untreated dairy products is a known way of transmission of TBEV. Milk-borne transmission for instance was common in the Czech Republic in the 1950s, and the related disease was called ‘biphasic milk fever’ [7]. Since then, milk-borne TBE outbreaks have been reported in a number of central and eastern European countries [8-13]. One TBE outbreak caused by goat cheese happened in 2008 in a mountainous region in Austria [14].

In Germany, TBE has been a reportable disease since 2001, with some 200 to 400 human cases reported annually [15]. Most human cases in Germany appear in southern Germany, where the main known endemic areas are located. Only about half of the patients in Germany remember tick bites as the probable cause of the TBEV infection. However, so far, there has not been any evidence of milk-borne TBEV infection in Germany.

We investigated a cluster of two human TBE cases that occurred in the spring/summer of 2016 in southern Germany in consumers of raw goat milk and cheese from a goat farm. After the clinical diagnosis of meningoencephalitis and serological confirmation as TBE in both cases, the local public health office was informed and an outbreak investigation was initiated on 14 June in order to identify the source of infection and the extent of the outbreak. The following points were addressed: identification of additional human cases, identification of the natural focus of TBEV on the goat farm, seroprevalence of anti-TBEV antibodies in goat sera on the farm and isolation and characterisation of TBEV from goat cheese and goat milk as well as ticks sampled around the farm.

## Methods

### Human infections

For active case finding among the consumers, information and an alert were released on 20 June 2016 by the local government through the regional local print media and by the manufacturer for their clients. The local public health office offered serological testing against TBEV by the German TBE consultant laboratory to all consumers who could be retrospectively identified. A human case was defined as exhibiting positive results in IgM and IgG against TBEV in association with the consumption of goat milk or cheese from the affected goat 4 four weeks before to 4 weeks after the first case was diagnosed and having no vaccination against TBE within the 6 months before. To exclude false positive results caused by cross-reactivity to other flaviviruses, all sera were tested for IgM and IgG antibodies against different flaviviruses (TBEV, West Nile virus, dengue virus, yellow fever virus, Japanese encephalitis virus) using an indirect immunofluorescence assay (Euroimmun, Lübeck, Germany), according to standard procedures. The consumers were asked for the date of the farm visit or cheese purchase, consumption date and total consumed amount, if available.

The regional physicians’ council was informed about suspected food-borne TBEV infections in the region. The Baden-Wuerttemberg State Health Office contacted all local health offices in the State to re-investigate already notified TBE cases with date of onset of clinical symptoms in May and June 2016 (calendar week 17–25), with special regard to consumption of raw goat milk products in the outbreak region within 21 days before disease onset.

### Goat cheese

The responsible food safety authority sampled unpasteurised goat cheese from the manufacturing and storage facilities of the dairy farm (16 June), and consumers provided available residual samples of cheese and milk produced between 5 May to 15 June by the goat farm. A 10% suspension in cell culture medium (Minimal Essential Medium, Invitrogen, Karlsruhe, Germany) was prepared and centrifuged and the ‘milk serum’, the watery phase, was used for nucleic acid extraction and further analyses. PCR-positive cheese samples were suspended in cell culture medium and incubated on 80% confluent Vero cells and A549 cells according to standard procedures of the Bundeswehr Institute of Microbiology. The cell culture supernatants were harvested on day 7 after inoculation and nucleic acid was extracted using the viral RNA extraction kit (Qiagen, Hilden, Germany). The extracted nucleic acids were tested by RT-qPCR according to a published protocol [16]. The E genes of TBEV were sequenced by Sanger sequencing as described in Kupca et al. [17].

### Goat flock

All goats in the flock were born on the farm. According to the owner’s information, no external animals from other farms had been added to the flock during the 2 years before the incident. For analysis of the goats’ serostatus and to find infected goats, we took a blood sample from each animal in the goat flock and tested it by virus neutralisation test at the German Federal Institute of Animal Health (Friedrich Loeffler-Institute, Greifswald-Insel Riems, Germany), using the low-pathogenic TBE group virus Langat virus according to standard procedures [18]. All sera were also tested for TBEV RNA by RT-qPCR [16] to demonstrate a potential acute viraemia in the animals.

### Ticks and natural foci

Because the meadows the goats used for grazing during the time before the outbreak (between April and May 2016) were documented by the farm owner, ticks were sampled around all suspected meadows between July and September 2016 by flagging along the vegetation. Ticks were identified and pooled according to stage and sex (three to 10 nymphs and two to five adult females or males per pool, all larvae in one pool). Pools were crushed using a MM400 mixer mill (Retsch, Germany) and three steel beads per pool, and nucleic acid was extracted using the EasyMag system (Biomerieux, Germany). Extracted nucleic acid was tested for TBEV RNA by a RT-qPCR [16].

## Results

### Human infections

**Figure 1:**
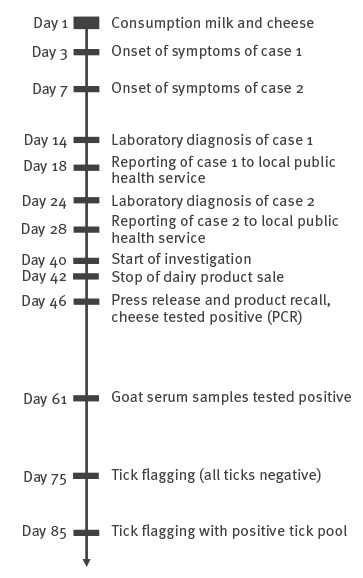
Investigation of a food-borne tick-borne encephalitis cluster Baden-Württemberg, Germany, May–July 2016 (n = 2)

The two cases who developed TBE after the consumption of raw milk and cheese from the goat farm in May 2016 were not vaccinated (Figure 1). The first case was a man who presented to hospital in May 2016 with fever, headache and difficulty to walk, 3 days after returning from a hiking holiday in south-west Germany with three other participants (one man, two women). They had visited a dairy farm where the first case and one other member of the group consumed unpasteurised goat milk and unpasteurised goat cheese. The second case was the other man from the same group who presented to hospital 4 days later with neurological symptoms.

During the course of illness, both cases developed various neurological symptoms and suffered from severe fatigue and weight loss. Both were admitted to hospital and treated for meningoencephalitis with empirical antibiotic and anti-viral therapy. Other potential causes were excluded by a number of different blood tests, cerebrospinal fluid tests and magnetic resonance imaging. TBEV serology was positive for IgM and IgG in the serum and cerebrospinal fluid (CSF) for both cases (day 14, day 24) (Figure 1). The clinical symptoms improved, but both continued to experience asthenia, neurological and psychiatric symptoms after 1 year. The final outcome is unfortunately unknown.

The two other hiking group members did not consume any milk and only one woman tried small amounts of the goat cheese but disgorged it immediately. None of the two women developed any neurological symptoms, nor IgM or IgG antibodies against TBEV. The farm owner reported more than 20 visitors/consumers on the assumed day of infection. Farm visitors consumed ca 20–30 glasses of raw milk and a much larger amount of cheese products. However, no further cases were reported from this potential exposure. After the second reported TBE case from the same family the district public health office started an investigation on day 40 resulting in a stop of the farm’s dairy product sale and all dairy products still available were recalled on day 42 (Figure 1). On day 46, the goat cheese samples tested positive and after two tick sampling activities on days 75 and 85, a positive tick pool was detected by PCR in the samples from day 85 (Figure 1).

None of 29 other consumers in June who responded to the press release of the manufacturer and the local government on day 46 after start of symptoms of the first human case (Figure 1) nor the dairy worker showed IgM antibodies against TBEV 3–8 weeks after consumption. Four consumers had low anti-TBEV IgG titres (without cross-reactions to other tested flaviviruses), which indicate either TBE vaccination or past infection.

The dairy worker had a high IgG titre but reported no TBE vaccination and no TBE-specific symptoms in the months before the event. The person remembered only a few tick bites in their life. As the dairy worker reported daily consumption of self-produced cheese but did not drink any goat milk, their serological result may be interpreted as a potential recent infection through cheese, although we cannot exclude an infection through ticks in the TBE focus area. The farm owner had been vaccinated against TBE early in the season 2016 and was therefore not tested. None of the 27 TBE cases notified until June 2016 in the State reported consumption of raw goat milk or cheese or a visit to the suspected farm; they were therefore not considered to be related to the outbreak.

### Dairy products

We tested 22 cheese samples and one milk sample from 18 different batches (production days) in May and June 2016 for the presence of TBEV RNA. Five samples from five different batches of cheese (cream cheese, soft cheese and ripened cheese) produced between 8 June and 12 June tested positive by repeated RT-qPCR. Two of the five samples could be confirmed by cell culture, indicating the viability of TBEV in the cheese (Table).

**Table:** Goat milk products tested for the presence of tick-borne encephalitis virus, Baden-Württemberg, Germany, May to June 2016 (n = 23)

Food item (unpasteurised)	Production date	PCR	Culture
Goat milk	16 Jun 2016	Negative	ND
Goat cream cheese	15 Jun 2016	Negative	ND
Goat cream cheese	14 Jun 2016	Negative	ND
Goat cream cheese	13 Jun2016	Negative	ND
Goat cream cheese	12 Jun2016	**Positive**	**Positive**
Goat cream cheese	11 Jun2016	Negative	ND
Goat cream cheese	10 Jun2016	**Positive**	**Positive**
Goat cheese, ripened	9 Jun2016	**Positive**	Negative
Goat soft cheese	8 Jun2016	**Positive**	Negative
Goat soft cheese	5 Jun2016	Negative	ND
Goat soft cheese	6 Jun2016	Negative	ND
Goat cheese, ripened	4 Jun2016	Negative	ND
Goat cream cheese	Jun 2016^a^	**Positive**	Negative
Goat cream cheese with curry	Jun 2016^a^	Negative	ND
Goat soft cheese	Jun 2016^a^	Negative	ND
Goat soft cheese	Jun 2016^a^	Negative	ND
Goat soft cheese	May 2016^a^	Negative	ND
Goat soft cheese	May 2016^a^	Negative	ND
Goat soft cheese	May 2016^a^	Negative	ND
Goat soft cheese	May 2106^a^	Negative	ND
Goat soft cheese	May 2016^a^	Negative	ND
Goat soft cheese	3 May 2016^b^	Negative	ND
Goat soft cheese	April 2016^a^	Negative	ND

ND: not done.

^a^ Stored cheese with unknown production date.

^b^ Selling day at the dairy farm.

We retested by TBEV RT-qPCR the five PCR-positive cheese samples each divided into 10 portions of ca 1 g. Three and two portions from the two culture-confirmed samples were weakly positive, while the remaining 45 samples were negative, indicating inhomogeneous distribution of virus in the cheese samples. 

**Figure 2:**
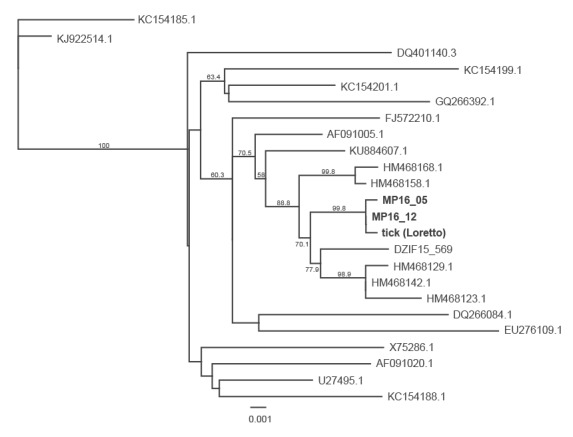
Phylogeny of the E genes of a tick-borne encephalitis virus isolated from goat cheese compared with related and reference TBEV strains, Baden-Württemberg, Germany, 2016 (n = 22)

The five positive samples were inoculated in cell cultures. Two of the samples yielded a virus isolate in Vero and A549 cells. Sub-passages were performed and the E genes of the isolated virus strains were amplified and sequenced. Both isolates had 100% identical nucleotide sequences for the E genes. Using the maximum likelihood method, the closest phylogenetic relation was found to the E genes of a TBEV strain we isolated from a tick pool in 2015 in Pitztal, Austria, and to published sequences from different locations in Switzerland (Zofingen, Dagmarsellen, Brittnau, Lommis, Schattdorf) (Figure 2) [19]. 

Sequences were chosen to include the reference strains Neudörfl and Hypr and the genetically closely related strains to demonstrate the genetic relatedness to the strains. E gene sequence accession numbers and origin of isolates: TBEV strain MP16_05 (GenBank accession number MH704556*) and TBEV strain MP16_12 (GenBank accession number MH704555*): goat cheese isolates (this study, in bold); TBEV E gene sequence tick (Loretto, GenBank accession number MH704557*): tick sequence (this study, in bold); KC154185.1: TBEV strain HB171-11 (Germany 2011); KJ922514.1: TBEV strain Skrivanek (Czech Republic, 1953); DQ401140.3: TBEV strain Torö (Sweden, 2003); KC154199.1: TBEV strain A104 (Austria, 1990); KC154201.1: TBEV strain CG-223 (Slovak Republic, 1990); GQ266392.1: TBEV strain AS33 (Germany 2005); FJ572210.1: TBEV strain Salem 2008); AF091005.1: TBEV strain Absettarov (Russia, 1951); KU884607.1: TBEV strain Leila-BH95/15 (Germany, 2015); HM468168: TBEV strain UR Schattdorf1 (Switzerland, 2010); HM468158.1: TBEV strain TG Lommis1 (Switzerland, 2010); DZIF 15_569: TBEV strain Pitztal (Austria, 2015); HM468129.1: TBEV virus strain AG Zofingen (Switzerland, 2010); HM168142.1: TBEV strain LU Dagmarsellen (Switzerland, 2010); HM468123.1: TBEV strain AG Brittnau (Switzerland, 2010); DQ266084.1: TBEV strain 263/5 (Czech Republic, 1991); EU276109.1: TBEV strain KrM93 (South Korea, 2006); X25286.1: TBEV strain Hypr (Czech Republic, 1953); AF091020.1: virus strain ZZ9 (Austria, 1985); U27495.1: TBEV strain Neudörfl (Austria, 1971); KC154188.1: TBEV strain HM377-11 (Germany, 2011).

### Goat flock

Blood was taken from all 45 goats at the beginning of June 2016 and tested for TBE antibodies. The animals were 1–11 years-old. Nine goats had neutralising antibody titres > 15 against TBEV, which proved that these animals had been infected with TBEV in the past. Two of the tested goats had high neutralising antibody titres ≥ 120. None of the animals were viraemic at the time of testing as all RT-qPCR were conducted according to the standard diagnostic procedures and results from all sera gave negative results.

### Ticks and natural foci

A total of 424 ticks were collected in three sampling activities in July, August and September 2016 along the meadows and in a forest from the vegetation along the daily pathway of the goats from the meadows to the stables. All ticks were morphologically identified as *Ixodes ricinus.* The single larval pool (17 larvae), the 33 nymphal pools (total 237 nymphs) and all 24 adult male pools (total 81 males) gave negative results in the TBEV RT-qPCR. One of 23 adult female pools (total 89 female ticks), which was sampled on 22 July 2016 and contained two adult females, was PCR-positive for TBEV. Testing the ticks from this pool individually, showed one adult female to be positive for TBE viral genome. An overall infection rate of 0.24% (1/424) was determined in the local tick population. A phylogenetic comparison of the E genes from the cheese isolates with the E gene sequence of the positive tick showed almost identical sequences. Only 2 of 1,433 nt were different and did not entail amino acid changes (Figure 2).

## Discussion

Milk-borne TBE outbreaks have been reported since the 1950s in Europe. Transmission of TBEV by milk of goats, sheep and cows is known and was observed in recent decades mainly in central and eastern European countries [7-14]. In Germany, food-borne transmission of TBEV has so far not been reported and is not regarded as epidemiologically important [20]. We report here the simultaneous occurrence of two cases of TBE transmitted by raw goat milk or cheese in Germany. A specific goat farm in south-west Germany could be identified as the location of infection. Although tick bites as source of infection could not be excluded, none of the two cases reported any ticks or tick bites on the suspected day of infection Furthermore, the probability of two concurrent TBE cases occurring during one stay in a TBE focus, assuming an average TBEV prevalence rate in ticks of 0.5%, is minimal.

This cluster of milk-borne TBEV outbreak in Germany had some unusual characteristics. None of more than 20 other guests of the goat farm who were exposed on the same day to the same batches of cheese and milk presented any neurological symptoms resembling TBE. In all milk-borne outbreaks described so far, the manifestation index among exposed and infected persons was high [8-14]. The infectious dose for infection of humans via the oral route by dairy products is unknown. One possible explanation could be that only small portions of the milk or cheese on the goat farm contained enough TBEV to cause human infection. Our RT-qPCR and isolation results led us to conclude that TBEV was inhomogeneously distributed in the goat cheese, which implies that the overall amount of virus in the investigated cheese was small, maybe at the lower range of the oral infectious dose for humans. As we have some indication of infectious raw milk and raw milk cheese from May (human cases) and June (cell-culture positive cheese), it is noteworthy that no other human TBE cases could be linked to the consumption of the products.

It is known that low-pathogenic and non-pathogenic TBEV strains circulate in ticks in nature [21]. Therefore, the TBEV isolates from milk and ticks will offer a unique opportunity to study phenotypical and pathogenetic characteristics so as to find first evidence on whether passage through the goat and shedding by goat milk could change the genotype and pathogenicity of TBEV. Data on the pathogenesis of TBE in the gastrointestinal tract are not yet available. It remains unclear where exactly the oral TBEV infection will take place, in the upper or lower gastrointestinal tract. In the latter case, it needs to be studied how TBEV survive the gastric passage with its acid environment.

Our interpretation of the serological results is that one or both goats with the high levels of neutralising TBEV antibodies may have been the source of the infections and might have shed TBEV with milk at the time of infection of the two cases. We do not have any data on the shedding of TBEV in different goat breeds. It is also unknown whether different TBEV strains exhibit different shedding rates in goats. One explanation of the low virus content in the milk products might be that the goat breed in question did not shed large amounts of TBEV in the milk or that the local TBEV strain might not replicate well in goats. We cannot exclude that one or more goats that were shedding virus were re-infected and therefore shedding only small amounts of TBEV in milk. So far, information is scarce on the duration of immunity in goats after TBEV infection, re-infection of previously infected animals and shedding under such circumstances, although one study has suggested immunity in goats after natural infection and vaccination [18]. Recent data demonstrated a decrease in antibodies against TBE in experimentally infected goats within 1 year [22]. Only systematic experimental infection of different goat breeds can test this hypothesis.

The detection of TBEV in a tick from the meadows of the farm demonstrates that TBEV circulated in ticks in the region in 2016. The serological data from the goats imply that some goats had been infected a longer time ago, while two goats with high anti-TBE antibody titres may have had a recent infection. The data suggest that TBEV infection in goats may occur and confirm earlier results from southern Germany [4].

To the best of our knowledge, this is the first time that TBEV could be isolated from cheese of a naturally infected goat after a cluster of TBE with transmission through goat milk. It is so far unclear whether the passage through the goat and the shedding via milk may select for virus strains with changed pathogenicity by the oral route. Our isolates from cheese and from a tick will allow addressing this question by sequencing and analysis of the complete genome of the isolates in ongoing and future studies.

Phylogenetic analysis of the E gene of one of TBEV isolates from goat cheese showed that the virus formed a genetic cluster with TBEV strains from different locations in Switzerland and Austria. This result may support the working hypothesis of discontinuous recent spread of TBEV over long distances, maybe by birds or by human activities (e.g. passive transport by pet animals).

During the 5 years before our cluster (2011–15), 608 human TBE cases were notified in the State of Baden-Württemberg. Only 11 cases were reported from the district where the goat milk transmission occurred (incidence < 1:100,000 population) and no cases were reported from the community to which the goat farm belongs. Ca 30–50% of all notified human TBE cases in Germany do not remember a tick bite before the disease. It has to be studied in more detail whether some of these cases may have been caused by food-borne transmission. We therefore recommend that public health officials and physicians consider this route of infection in the future.

Recent laboratory findings showed that TBEV may survive in processed milk within the food chain [23]. Therefore, the risk of contamination of raw milk products ready for consumption should be reassessed. Until then, consumer protection authorities should provide tailored messages to communicate the risk. This milk-borne TBE cluster in Germany shows the potential of TBEV spread and emergence also in a small local manufacturers even in countries with highly industrialised food production and again illustrates our deficits in the knowledge of TBEV pathogenicity and eco-epidemiology.

## *Addendum

The GenBank accession numbers were added on 1 October 2018 in the note to Figure 2.
