# Addendum for Euro Surveill. 2018;23(15)

**DOI:** 10.2807/1560-7917.ES.2018.23.40.181004

**Published:** 2018-10-04

**Authors:** Eurosurveillance editorial team

**Affiliations:** 1 European Centre for Disease Prevention and Control (ECDC), Stockholm, Sweden

In the article entitled ‘A cluster of two human cases of tick-borne encephalitis (TBE) transmitted by unpasteurised goat milk and cheese in Germany, May 2016‘ by SO Brockmann et al., published on 12 April 2018, GenBank accession numbers were added on 1 October 2018 at the request of the authors.

